# Bladder Hyperactivity Induced by Oxidative Stress and Bladder Ischemia: A Review of Treatment Strategies with Antioxidants

**DOI:** 10.3390/ijms22116014

**Published:** 2021-06-02

**Authors:** Yi-Hsuan Wu, Kuang-Shun Chueh, Shu-Mien Chuang, Cheng-Yu Long, Jian-He Lu, Yung-Shun Juan

**Affiliations:** 1 Graduate Institute of Clinical Medicine, College of Medicine, Kaohsiung Medical University, Kaohsiung 80708, Taiwan; maivy0314@gmail.com (Y.-H.W.); spacejason69@yahoo.com.tw (K.-S.C.); 2 Department of Urology, College of Medicine, Kaohsiung Medical University, Kaohsiung 80708, Taiwan; u9181002@gmail.com; 3 Department of Urology, Kaohsiung Municipal Hsiao-Kang Hospital, Kaohsiung 80661, Taiwan; 4 Department of Urology, Kaohsiung Municipal Ta-Tung Hospital, Kaohsiung 80145, Taiwan; 5 Department of Obstetrics and Gynecology, Kaohsiung Medical University Hospital, Kaohsiung 80708, Taiwan; urolong@yahoo.com.tw; 6 Regenerative Medicine and Cell Therapy Research Center, Kaohsiung Medical University, Kaohsiung 80708, Taiwan; 7 Emerging Compounds Research Center, Department of Environmental Science and Engineering, College of Engineering, National Pingtung University of Science and Technology, Pintung 91201, Taiwan; toddherpuma@yahoo.com.tw

**Keywords:** overactive bladder, oxidative stress, bladder dysfunction, antioxidants

## Abstract

Overactive bladder (OAB) syndrome, including frequency, urgency, nocturia and urgency incontinence, has a significantly negative impact on the quality-of-life scale (QoL) and can cause sufferer withdrawal from social activities. The occurrence of OAB can result from an imbalance between the production of pro-oxidants, such as free radicals and reactive species, and their elimination through protective mechanisms of antioxidant-induced oxidative stress. Several animal models, such as bladder ischemia/reperfusion (I/R), partial bladder outlet obstruction (PBOO) and ovarian hormone deficiency (OHD), have suggested that cyclic I/R during the micturition cycle induces oxidative stress, leading to bladder denervation, bladder afferent pathway sensitization and overexpression of bladder-damaging molecules, and finally resulting in bladder hyperactivity. Based on the results of previous animal experiments, the present review specifically focuses on four issues: (1) oxidative stress and antioxidant defense system; (2) oxidative stress in OAB and biomarkers of OAB; (3) OAB animal model; (4) potential nature/plant antioxidant treatment strategies for urinary dysfunction with OAB. Moreover, we organized the relationships between urinary dysfunction and oxidative stress biomarkers in urine, blood and bladder tissue. Reviewed information also revealed the summary of research findings for the effects of various antioxidants for treatment strategies for OAB.

## 1. Overactive Bladder (OAB)

The International Continence Society (ICS) defined overactive bladder (OAB) as urinary urgency, usually accompanied by frequency and nocturia, urgency with or without urgency urinary incontinence, in the absence of urinary tract infection or other obvious pathology [1,2]. However, detrusor overactivity (DO) is another condition. The ICS defined DO as spontaneous or provoked involuntary detrusor contractions during filling cytometry of urodynamic study. OAB has been linked to DO, but not every OAB patient has DO. In a prospective study, only 64% of OAB patients had DO. In addition, in patients who had DO on filling cystometry, 30% had no OAB symptoms.

A recent study investigated the population of Eastern-European countries. The prevalence of lower urinary tract symptoms (LUTS) was 66.2% in men and 72.6% in women. Moreover, the prevalence of OAB was higher in women (39.5%) than in men (26.8%) and increased with age. Of interest, women were more likely to be bothered in comparison with men [1]. In Asian countries, a questionnaire-based epidemiology study from 11 countries showed that the prevalence of OAB was 53.1% in women and 29.9% in men [3]; in men aged over 70 years, this increased to 53% [4,5]. In Taiwan, human epidemiology studies revealed that the prevalence of female OAB was 20.9%, with an age-dependent increase to 34.5% in those over the age of 65 years [6,7]. Symptoms of OAB have a significant negative impact on the quality of life (QoL) and can withdraw the sufferer from social activities. Moreover, increased voiding times at night may cause significant sleep disturbances, resulting in fatigue and depression. In particular, urgency incontinence is related to an increase in the number of falls and fractures in the elderly population [8].

## 2. Pathophysiology of OAB and Its Relation with Oxidative Stress

Bladder storage and voiding depend on the interaction and synergy between efferent sympathetic, parasympathetic, somatic and afferent sensory nerves. Bladder oversensitivity and overactivity arise from several different conditions involving the bladder, bladder outlet and nervous system. In addition to nerve innervation, the detrusor muscle, the urothelium, the suburothelium and pelvic blood vessels all play an important role in the pathophysiologies of OAB. Parasympathetic nerves trigger the contraction of the bladder detrusor muscle through the stimulation of both M2 and M3 muscarinic receptors by acetylcholine and the purinergic receptor (P2X) by ATP, which also relax the urethral smooth muscle through the action of nitric oxide (NO). ATP and purinergic receptors are involved in modulating urgency symptoms and the micturition pathway in urological diseases. Activation of the adrenergic receptor (such as AR-α and AR-β) by catecholamines can facilitate the micturition reflex. AR-β stimulation activates the adenylyl cyclase pathway in bladder urothelium and initiates an increase in intracellular Ca^2+^, which triggers NO production and release. In addition, the somatic pudendal nerve stimulates the striated muscle of the external urethral sphincter through nicotinic receptor stimulation, and sympathetic receptors expressed in human detrusor and urothelium are α1-adrenoreceptor (α1-ARs) and β- adrenoreceptor (β-ARs).

The pathophysiology of OAB is composed of multiple possible causes that are not fully elucidated. Chu et al. classified OAB into neurogenic OAB (e.g., spinal cord injury), myogenic OAB (e.g., bladder outflow obstruction), inflammatory OAB (e.g., interstitial cystitis) or idiopathic OAB [9]. The mechanism for OAB may be related to bladder innervation, muscarinic (such as M2 and M3) and purinergic (such as P2X3) receptors and an abnormal increase in the production of cyclooxygenase-2 (COX-2), prostaglandin and leukotriene [10,11]. For example, overexpression of urothelial transient receptor potential vanilloid 1 (TRPV1) [12] and P2X3 receptors [13] and hypersensitivity of the C-fiber pathway are associated with urgency and DO in humans [14]. Changes in urothelial receptor function and neurotransmitter release, as well as changes in the sensitivity and coupling of the suburothelial interstitial cells, may lead to enhancement of involuntary bladder contractions [15]. In addition, in streptozocin-induced diabetic rats, OAB is associated with increased ATP and decreased constitutive NO release from the urothelium [16].

Previous studies also revealed that hypoxia, excessive oxidative stress and loss of blood supply play a pivotal role in OAB [17,18]. The bladder needs blood supply for oxygen and nutrition for normal storage and voiding function. Decrease in blood saturation induces hypoxia, accompanied by an abundance of oxidative free radicals and the subsequent impairment of bladder contractility and compliance. During the filling phase, the bladder wall maintains a high level of oxygen saturation. However, blood vessels are compressed, and bladder wall blood flow is reduced during bladder emptying. Therefore, cumulative oxidative stress caused by bladder cyclic I/R leads to LUTS, including OAB [18,19].

## 3. Medical Management of OAB

Medical treatment targeting of OAB might relieve overactive bladder symptoms in a portion of patients. Currently, the medical treatment of OAB consists of antimuscarinic agents or/and β3 agonist medication. Given the involvement of the parasympathetic system in the lower urinary tract, the standard therapy for OAB is represented by antimuscarinics, which are not selective for bladder receptors, with some systemic side effects, such as constipation, dry mouth and blurred vision. A previous study showed that half of patients stopped antimuscarinic treatment within 150 days of treatment due to intolerable adverse effects, and only 8.3–24% of patients could maintain long-term usage [20]. In 2012, the FDA approved the first β3 agonist, mirabegron, to treat patients with OAB symptoms. The β3 agonist works via the sympathetic nerve pathway and stimulates beta-3 receptors to relax the bladder smooth muscle. Side effects of the β3 agonist include somnolence and unstable blood pressure, which make people with OAB unwilling to continue medication [21]. Severe adverse effects, such as retention of urine and urinary tract infection (UTI), significantly increase with age [22].

Whenever medical treatment failed, invasive intervention with botulinum toxin A (BoNT-A) bladder injection and tibial nerve stimulation was considered [23,24]. BoNT-A treatment of OAB has anti-inflammatory and antinociceptive effects to relieve OAB symptoms and increase functional bladder capacity. In patients with DO treated with detrusor BoNT-A injections, the expressions of TRPV1 and P2X3 receptors were reduced on the suburothelial sensory afferents [25]. Therefore, BoNT-A therapy for OAB has shown significant improvement in urinary urgency, bladder pain and bladder capacity. However, this invasive procedure may result in side effects, including hematuria, increased residual urine, catheter drainage risk, nerve pain and urinary tract infections. Recently, OAB was shown to be relieved with low-intensity extracorporeal shockwave therapy (LiESWT). LiESWT could improve the voided volume and ameliorate OAB symptoms, including frequency, urgency, nocturia and urinary incontinence. Significant improvement in social activity and QoL suggests that LiESWT might be a future alternative treatment for OAB [26].

## 4. Oxidative Stress and the Antioxidant Defense System

Oxidative stress is related to increased intracellular levels of reactive oxygen species (ROS), reactive nitrogen species (RNS) and free radicals, such as superoxide anion radical (O_2_^•−^) and hydroxyl radicals (^•^OH), as well as nonradicals, such as H_2_O_2_, nitric oxide (NO), peroxynitrite, and hypochlorous acid. ROS are derived from oxidant enzymes, such as nicotinamide adenine dinucleotide phosphate (NADPH), xanthine oxidase, cyclooxygenases (COX) and the mitochondrial respiratory chain. Mitochondria produces chemical energy that is stored in adenosine triphosphate (ATP) through the oxidative phosphorylation process. However, damaged mitochondria produce excess ROS and result in rapid depolarization of mitochondrial inner membrane potential and impairment of oxidative phosphorylation. For example, H_2_O_2_ is generated from superoxide produced by NADPH oxidases, the mitochondrial respiratory chain and diverse oxidases [27,28,29]. Low and moderate concentrations of ROS/RNS are useful for ordered cellular signaling and mitogenic responses. Therefore, the balance between ROS generation and ROS scavenging was highly controlled under physiological conditions. Unregulated oxidative and reductive stresses could result in cell damage, cell death and consequently organ failure.

## 5. Redox Signaling and the Nrf2/ARE Pathway

Adaptive physiological redox signaling is essential for the maintenance of homeostasis between oxidants (ROS generation) and antioxidants (ROS elimination); however, excessive ROS/RNS production leads to DNA damage, protein adducts, lipid peroxidation and mitochondrial dysfunction and results in various pathological conditions, including cancers, diabetes and cardiovascular diseases. Nocchi et al. found that oxidative stress is related to increased bladder nerve activity and intravesical pressure with H_2_O_2_ [30].

Cellular defense against oxidative stress was activated by the Nrf2-antioxidant response element (ARE) signaling pathway to control the translated expression of genes involved in the detoxication and elimination of reactive oxidants by promoting antioxidant capacity [31]. The Keap1/Nrf2 stress response pathway is the inducible protective response against oxidative stress through the regulation of cytoprotective gene expression. Under homeostatic conditions, KEAP1 forms part of an E3 ubiquitin ligase to regulate the expression of Nrf2 by ubiquitination and proteasome degradation. However, during the stimulation of excessive oxidative stress, KEAP1 facilitated by cysteine oxidation assisted Nrf2 to get away from cellular ubiquitination, translocated into the nucleus and integrated with AREs to promote the expression of downstream genes, including phase II detoxifying enzymes (heme oxygenase-1 (HO-1), glutamate-cysteine ligase (GCL)), NADPH and antioxidant enzymes (SOD, CAT and GSH-Px) to inhibit oxidative stress production [32,33]. The Keap1/Nrf2 pathway promoted antioxidant transcription response and played a critical regulatory role in various pathological conditions induced by oxidative stress, including cancers, chronic inflammation, neurodegenerative and cardiovascular diseases [34]. The protective role of Nrf2 was attributed to antioxidant and phase II detoxifying reactions to facilitate antioxidant capacity [35]. Transcriptional control of the phase II enzymes was mediated through ARE found in the regulatory region of phase II genes encoding phase II enzymes ARE [36]. Taken together, Nrf2 is maintained at a low level via KEAP1-mediated proteasome degradation. In response to phase II inducers, the constitutive degradation of Nrf2 was inhibited, increased cellular Nrf2 accumulation, then translocated to the nucleus to regulate cytoprotective gene expression.

## 6. Antioxidant Defense Systems

Organisms have developed antioxidant defense systems to eliminate ROS. Cellular ROS levels are regulated by an enzymatic antioxidant system. The defense systems include enzymes such as superoxide dismutase (SOD), catalase (CAT) and glutathione peroxidase (GSH-Px). SOD can transform superoxide into H_2_O_2_, and then, H_2_O_2_ is catalyzed by CAT and GSH-Px [37]. SODs prevent the accumulation of superoxide to damaged tissue and inactivate proteins containing iron–sulfur clusters [38]. SOD1 is mainly located in the cytosol and mitochondrial intermembrane space, SOD2 is located in the mitochondrial matrix and SOD3 is located in the plasma membrane [39,40]. SOD and CAT activities are associated with the shift from the compensated to decompensated function of the bladder [39].

## 7. Oxidative Stress in OAB and Biomarkers of OAB

Generally, OAB is a symptomatological description. Therefore, the diagnosis of OAB needs the biomarkers to predict treatment success. The clinical value of urinary biomarkers in the objective and noninvasive assessment of patients with OAB symptoms and therapeutic outcomes remains unknown. Currently, putative biomarkers include urinary and serum nerve growth factor (NGF), urinary brain-derived neurotrophic factor (BDNF), urinary cytokines, urinary prostaglandin E2 (PGE2) and serum C-reactive protein (CRP) [41]. These biomarkers are related to nerve growth and/or inflammation.

In OAB studies, pathological activities and regulative mechanisms of oxidative stress are extremely complex, and much of them are unknown. The pathological significance of oxidative stress in the bladder is important to understand the potential treatment strategies for patients with urinary dysfunctions. A previous study suggested that activation of the Nrf2/ARE pathway may ameliorate bladder dysfunction caused by bladder outlet obstruction (BOO) [42]. Mice with a mutation in the Immp2l gene can lead to high superoxide ion production [43], leading to bladder void dysfunction [44]. Mutant mice with increased detrusor activity that have a higher bladder to body weight ratio could be a model of oxidative stress to provide a tool to study the role of oxidative stress on bladder function [45].

Changes in the biomarkers of oxidative stress in OAB animal models support the association between oxidative stress and urinary dysfunction. Biomarkers of oxidative damage such as protein carbonyls, lipid peroxidation products or breakdown products of damaged DNA are often used [46]. The oxidative stress markers involved in OAB include 8-hydroxy-2′-deoxyguanosine (8-OHdG), malondialdehyde (MDA) and isoprostanes (IsoPs).

Table 1 summarizes the oxidative stress biomarkers in urine, blood and tissue samples, and their preliminary data in clinical trials of animal models are described below:

### 7.1. 8-Hydroxy-2′-Deoxyguanosine (8-OHdG)

8-OHdG is the predominant form of free-radical-induced oxidative lesions and has been widely used as a biomarker for oxidative stress, aging, diabetes and carcinogenesis both in human and animal studies. Increased levels of 8-OHdG may be due to damaged nuclear and mitochondrial DNA as a result of oxidative attacks caused by free radicals [50]. In PBOO animal models, changes in urinary levels of 8-OHdG support the association between oxidative stress and urinary dysfunction.

### 7.2. Malondialdehyde (MDA)

MDA is a physiologic ketoaldehyde produced by the peroxidative decomposition of unsaturated lipids as a byproduct of arachidonate metabolism. MDA has been used as a marker to assess oxidative stress and degree of tissue destruction [37], where the MDA concentration was significantly higher in the OAB [50]. Matsui et al. [64] revealed that the MDA level was increased in a rat model of atherosclerosis-induced chronic bladder ischemia and enhanced oxidative stress. In PBOO rabbits, there was a significant increase in bladder weight and the levels of urine 8-OHdG and plasma MDA, while there was a significant decrease in the total antioxidant capacity (TAC) in plasma compared to sham rabbits [48,51].

### 7.3. Isoprostanes (IsoPs)

IsoPs are classified as prostaglandin isomers and major oxidative stress markers because they are end products of lipid peroxidation stimulated by free radicals [37]. In addition, F2-isoprostanes (F2-IsoPs) are chemically stable metabolic products produced by ROS, which are detectable in tissues, but not in urine or blood [63,65]. F2-IsoP levels in bladder tissues of a PBOO mouse were mildly increased immediately after bladder distention [62]. Dambros et al. [66] used hypochlorous acid to induce pig bladder overactivity and found that 8-iso-PGF2α levels were increased in the overactive group, and the level of overactivity was correlated with oxidative stress.

## 8. OAB Animal Model

**Figure 1:**
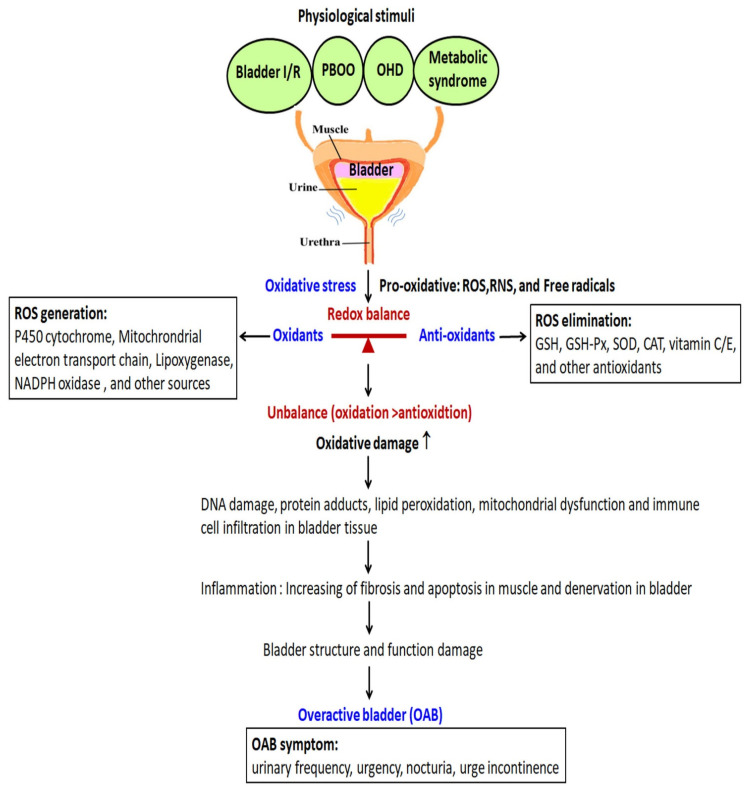
Disorders associated with oxidative stress and overactive bladder (OAB), including bladder I/R, PBOO, metabolic syndrome, and ovary hormone depletion (OHD). OAB syndrome includes frequency, urgency, nocturia and urgency incontinence. The occurrence of OAB may result from an imbalance between the production of pro-oxidants and their elimination through antioxidants. Several animal models mimic the physiological condition to induce OAB, such as bladder ischemia/reperfusion (I/R), partial bladder outlet obstruction (PBOO), metabolic syndrome and ovarian hormone deficiency (OHD). Oxidative stress is regulated by the balance between pro-oxidative and antioxidative factors. Oxidative stress increased the levels of cellular reactive oxygen species (ROS), reactive nitrogen species (RNS) and free radicals. The antioxidant defense system participates in scavengers of free radicals to decrease excessive ROS and protect against oxidative-stress-induced bladder dysfunction. Redox signaling is essential for the maintenance of homeostasis between oxidants (ROS generation) and antioxidants (ROS elimination). ROS are derived from nicotinamide adenine dinucleotide phosphate (NADPH) oxidase (NOX), xanthine oxidase, nitric oxide synthase (NOS) and cyclooxygenases’ mitochondrial respiratory chain. Cellular ROS levels are regulated by an enzymatic antioxidant system. The defense systems include enzymes such as superoxide dismutase (SOD), catalase (CAT) and glutathione peroxidase (GSH-Px). However, excessive production of ROS/RNS can cause oxidative stress, which results in DNA damage, protein adducts, lipid peroxidation, mitochondrial dysfunction and immune cell infiltration in bladder tissue leading to bladder denervation and increasing fibrosis and apoptosis, resulting in bladder hyperactivity.

Many disorders have confirmed the association between oxidative stress and bladder dysfunction, including metabolic syndrome, obesity, diabetes, bladder I/R, BPO, PBOO, and ovary hormone depletion (OHD) (Figure 1). In these OAB animal models, experts substituted objective urodynamic observation and nonmicturition contractions to evaluate the pathophysiology of OAB and to address the clinical challenges of OAB.

### 8.1. OAB in a PBOO Model

Clinically, more than 80% of men over 50 years old have some degree of PBOO and experience bladder problems as a result of enlarged prostates, urethral strictures or detrusor–sphincter dyssynergia [67]. PBOO results in changes in bladder structure and function that include detrusor hypertrophy, bladder wall thickness, elevated contractile pressure and detrusor instability leading to OAB [68,69,70]. Animal models of PBOO, in which the urethra is partially ligated, have been developed and have shown changes in morphology and function similar to that in humans [71,72]. According to the results of a variety of animal experiments on PBOO, there is a general agreement that ischemic bladder blood supply and repeated cyclic bladder I/R play a crucial role in the pathological mechanism of PBOO-induced bladder damage. In PBOO animal models, increasing the production of ROS strengthened MDA production and decreased antioxidant activities of CAT, GSH and SOD [37,73]. Biomarkers for oxidative stress in PBOO animal models linking the association between oxidative stress and urinary dysfunction are shown in Figure 1. Repeated cyclic bladder I/R and an increased level of ROS and free radicals in PBOO resulted in LUTS. Antioxidants could serve as potential therapeutic agents.

### 8.2. OAB in Chronic Ischemia and Ischemia/Reperfusion Bladder Model

Epidemiological studies have also shown a close association between LUTS and vascular risk factors for atherosclerosis. Investigations using transrectal color Doppler ultrasonography have shown a negative correlation between decreased lower urinary tract perfusion and International Prostate Symptom Score in elderly patients with LUTS [74,75]. Alexandre et al. [76] reported that chronic ischemia in the bladder enhances oxidative stress, leading to detrusor overactivity and storage symptoms and increased oxidative stress, and plays an important role in bladder dysfunction (Figure 1). To detect the influence of I/R on the bladder, Juan et al. [77] clamped the vesicle artery for 2 h then re-perfused the bladder. There are diminished contractile responses to electric and ATP stimulation and significant increases in several calcium-sensitive and smooth muscle tension regulatory proteins. Moreover, functional changes in bladder ischemia have been reported to be associated with impairment of mitochondrial respiration, cellular stress and activation of cell survival signaling via the PI3K/Akt pathway [78].

Evidence from clinical and animal research suggests that atherosclerosis can induce a reduction in bladder blood flow, leading to chronic ischemia of the bladder [79]. Matsui et al. [80] found that levels of both 8-OHdG and MDA were increased in a rat model of atherosclerosis-induced chronic bladder ischemia. Nomiya et al. also demonstrated that chronic vesicle atherosclerosis was associated with changes in oxidative stress markers (8-OHdG and MDA) and proinflammatory cytokines (TNF-α, and interleukin-6) in a rat model accompanied by bladder dysfunction [81]. The findings suggested that oxidative stress and inflammation may be essential factors in the development of bladder dysfunction in atherosclerosis-induced chronic bladder ischemia.

Improvement of lower urinary tract perfusion and control of oxidative stress can be considered a new therapeutic strategy for the treatment of bladder dysfunction induced by chronic ischemia [82]. The α1-adrenoceptor antagonist, phosphodiesterase type 5 inhibitor, free radical scavengers and the β3-adrenoceptor agonist have been studied in animal models of chronic bladder ischemia. Drugs that increased blood flow and decreased oxidative stress showed protective effects not only on urodynamic parameters but also on muscle contractility and on bladder wall changes.

### 8.3. OAB in Metabolic Syndrome (MetS) Model

MetS includes a cluster of cardiovascular disease risk factors, including obesity, dyslipidemia, hypertension, insulin resistance with compensatory hyperinsulinemia and glucose intolerance. MetS is not a single condition but a cluster of two or more of these five specific factors. Though the definitions of MetS are different among different guidelines, diagnosis of MetS mainly requires impaired glucose or fat regulation, raised blood pressure and central obesity [83]. Increasing clinical studies have revealed a strong association between MetS and OAB [84,85,86]. An elevated level of C-reactive protein (CRP) and some proinflammatory cytokines were detected in serum or urine of MetS patients, suggesting a role for systemic inflammation and oxidative stress in the pathophysiology of OAB [87,88]. However, the pathogenesis mechanism of MetS remains unknown.

Both oxidative stress and chronic inflammation play a pivotal role in the pathogenesis of MetS. Excessive production of superoxide anion (O2^•−^) and ROS/RNS in adipose tissues might cause the metabolic dysfunction of adipose tissue and result in bladder overactivity [89] (Figure 1). In obese mice, treatment with resveratrol or guanylyl cyclase activator BAY 60-2770 improved OAB via antioxidant activity. Obese mice exhibited bladder dysfunctions, such as increases in the frequency of voiding and nonvoiding contractions [76,90]. However, the correlation between obesity-associated OAB and oxidative stress is still not well studied.

Chronic hyperglycemia and insulin resistance could trigger superoxide (O2^•−^) overgeneration from the inner membrane of mitochondria [91]. Increased ROS production impaired mitochondria subsequently induced apoptosis by altering cellular redox potential [92]. In the fructose-fed rat model [93], MetS with hyperlipidemia and hyperglycemia resulted in ROS overproduction and impaired mitochondrial ATP production. Moreover, bladder purinergic and muscarinic signaling was altered after long-term fructose-induced MetS. Our previous study revealed that rats fed a high-fat high-sugar (HFHS) diet for 12 months could develop both MetS and OAB. In particular, MetS combined with surgical ovariectomy (OVX) worsens bladder storage dysfunction more intensely than MetS alone [94].

In the bladder, metabolic stress and inflammatory conditions can lead to increased production of nitro-fatty acids and NGF, which can activate transient receptor potential channels (TRP channels), thereby increasing bladder reflex activity. Similarly, high-fat diet-fed obese male mice showed increases in non-voiding contractions, post-voiding pressure and voiding frequency. The expression of oxidative stress markers (gp91phox and SOD1), ROS/RNS levels and serum lipid peroxidation in bladder tissues was meaningfully higher in obese mice compared with lean mice [95].

### 8.4. OAB in Menopause and Ovarian Hormone Deficiency (OHD) Model

Clinically, postmenopausal women, as a result of OHD, are subject to urological dysfunctions, including OAB symptoms, stress incontinence and recurrent urinary tract infections [96]. Up to 40% of postmenopausal women were estimated to have symptomatic urogenital atrophy [97]. The symptoms of postmenopausal women may have vulvovaginal atrophy, burning, dryness, discomfort, irritation, pelvic organ prolapse or pain [98]. Estrogen receptors have been shown in biopsy specimens from the bladder trigone, proximal urethra, distal urethra, vagina and vesicovaginal connective tissue contiguous with the bladder neck [99,100]. Estrogen deprivation in young patients with breast cancer increased the risk of OAB [101]. Without estrogen replacement, one-third of women experience symptoms of atrophic vaginitis, including dryness, irritation, itching and dyspareunia. Therefore, the efficacy of estrogen application for OAB, urinary urgency and stress incontinence during and after menopause has been demonstrated [102].

Experimentally, an OVX rat model mimicking the physiological condition of menopause was applied to induce OAB and investigate the role of estrogen in LUTS [103,104,105]. Our previous OVX rats investigation showed OHD resulted in diminishing bladder compliance, increasing oxidative damage, interstitial fibrosis and bladder mucosa cell apotosis [94] (Figure 1). OVX rats resulted in significant vascular degeneration and decreased vascular density, whereas estradiol administration mediated significant angiogenic remodeling characterized by increased vascular density and angiogenesis within the detrusor smooth muscle bundles. Both female genitals and lower urinary tract originating from the urogenital sinus are sensitive to sexual hormones. Alteration of sexual hormones results in bladder dysfunction in menopause, such as frequency, urgency, incontinence or underactive bladder [96,106]. On the other hand, estrogen supplement attenuates oxidative damage and improves bladder function [107,108]. Estrogen is essential for mediating physiologic functions in the female bladder.

## 9. Antioxidant Treatment in OAB

To restore the imbalance between oxidants and antioxidants in oxidative stress, organisms have evolved complex enzymatic defenses against the attacks of free radicals with the antioxidant defense [109]. Antioxidant defense systems involve scavengers of free radicals to neutralize excessive ROS and protect against bladder dysfunction caused by oxidative stress. Currently, several natural extract antioxidants, such as epigallocatechin-3-gallate (EGCG), coenzyme Q10 (CoQ10), melatonin, omega-3 fatty acid, Eviprostat and hydrogen water, have been used to improve bladder overactivity via inhibiting oxidative stress pathways (Table 2).

### 9.1. Epigallocatechin-3-Gallate (EGCG)

EGCG is a major component of green tea polyphenol. In addition, EGCG possesses radical scavenging activity and metal-chelating and anti-inflammatory properties. It has a therapeutic effect as an antioxidant compound to perform anti-inflammation, antifibrosis [116], anticancer, and antioxidative stress [117,118,119,120]. The phenol rings trap electrons and scavenge free radicals, preventing the formation of ROS [121,122]. EGCG can cause the removal of ROS, decrease oxidative stress and prevent the complications of diabetes by reducing the expression of proinflammatory cytokines [123]. EGCG was also reported to enhance the activity of antioxidant enzymes, superoxide dismutase (Mn-SOD and Cu/Zn-SOD) and CAT [124]. EGCG was considered a powerful hydrogen-donating antioxidant and a free-radical scavenger of ROS [104].

In OVX rats, EGCG has the ability to alleviate oxidative damage and bladder hyperactivity [104]. In BOO rats treated with EGCG, the level of MDA was significantly reduced. The activities of some redox status markers, such as CAT, GSH-Px and total SOD, were significantly decreased in the BOO group as compared with the sham group. These findings showed that EGCG could alleviate oxidative stress by increasing the activities of antioxidative enzymes in BOO rats. A previous report also revealed that treatment with EGCG significantly improved PBOO-induced histologic changes and bladder dysfunction and increased expressions of cyclooxygenase-2, poly (ADP-ribose) polymerase and ER stress markers such as caspase-12 and CCAAT/-enhancer-binding protein homologous protein (CHOP) [42].

The beneficial effect of EGCG is attributed to mitochondrial signal transduction in a concentration-dependent manner. Treatment with a low concentration of EGCG (1–10 μmoles) could restrain the proapoptotic caspase and raise the degradation of the Bax gene via the proteasome and protein kinase C pathway [125]. Additionally, treatment with a higher concentration of EGCG (10–50 μmoles) could cause caspase-dependent apoptosis and mitochondrial membrane depolarization to exhibit pro-oxidant and proapoptotic activity [126]. Coyleet et al. demonstrated that urothelial cell death via H_2_O_2_-induced oxidative stress was mediated through superoxide, treatment with EGCG can protect against bladder oxidative damage and urothelial cell death [127].

In our previous OVX-induced OAB rat animal study, supplementation with 10 μmoles of EGCG alleviated bladder apoptosis, attenuated oxidative stress and reduced the mitochondrial and endoplasmic reticulum apoptotic signals [103,104]. These results showed EGCG exhibited strong neuroprotective, antioxidant, antiapoptotic and antifibrotic effects in OVX-induced OAB [103,104]. Moreover, HFHS diet feeding enhanced the generation of oxidative stress mediated through the mitochondrial pathway. EGCG reduced the generation of oxidative stress and lessened bladder overactivity, including amelioration of the nonvoiding contraction and bladder compliance in OVX rats [94,103,104]. Moreover, EGCG treatment improved bladder inflammation via changes in apoptosis-related molecules such as Nrf2, caspase-3 and HO-1 [61]. The effect of EGCG ameliorated bladder dysfunction by inhibiting oxidative stress via the regulation of the Nrf2-ARE pathway to up-regulate HO-1 gene in a rat model of PBOO [61] The effect of EGCG ameliorates bladder dysfunction by inhibiting oxidative stress via the regulation of the Nrf2-ARE pathway to upregulate the HO-1 gene in a rat model of BOO [61].

### 9.2. Coenzyme Q10 (CoQ10)

Coenzyme Q10 (CoQ10; ubiquinone) is an essential molecule composed of a mitochondrial inner membrane [128]. The function of CoQ10 is to maintain the electrical gradient in mitochondria during ATP production. In addition, it helps the transport of electrons and protons and works as a cellular antioxidant, protecting cell membrane against oxidative damage [129]. Addition of exogenous CoQ10 recovered mitochondrial CoQ10 stores and increased the rate of electron transfer in the respiratory chain, thus improving the efficiency of oxidative phosphorylation and mitochondrial coupling and protection of creatine kinase. CoQ10 also appeared to be efficient for the treatment of neurodegenerative disorders and ischemic heart disease.

In a rabbit I/R model, CoQ10 supplementation upregulated SOD and CAT activities, provided bladder neuroprotection and decreased detrusor muscle apoptosis [111]. In a rat model, the efficacy of CoQ10 attenuated protein carbonylation and nitration to improve bladder function and histological changes in the bladder wall following chronic bladder ischemia and repeat I/R [112]. By analyzing the mitochondrial marker enzyme, citrate synthase and mitochondrial function were significantly increased after CoQ10 administration during I/R damage. In a PBOO model, CoQ10 supplementation exhibited both mitochondrial and neuronal effects; it not only ameliorated detrusor muscle hypertrophy but also reduced protein nitration and carbonylation to restore bladder dysfunction [113].

### 9.3. Melatonin

Melatonin, *N*-acetyl-5-methoxytryptamine, mainly secreted from the pineal gland, participates in many important physiological functions, including the control of the immune system, and the circadian rhythm [130]. Melatonin has a protective effect against oxidative stress and free radical agents and stimulates the activity of the endogenous antioxidant enzyme, GSH-Px. For example, melatonin is a potent antioxidant that suppresses oxidative stress caused by PBOO in animal models [56,57]. Bladder tissue levels of MDA decreased after treatment with melatonin in a PBOO model [57]. Tissue levels of CAT, GSH and SOD in PBOO rabbits treated with melatonin recovered to the levels in the sham group, whereas such significant effects were not detected following treatment with terazosin, an α1-adrenoreceptor antagonist [57]. In a rodent I/R model, lipid peroxidation, myeloperoxidase and MDA were elevated by ligation of the abdominal aorta. These oxidative activities could be reversed by treatment with melatonin, where the low contractility of the bladder was also improved [56]. The effect of melatonin as a potent antioxidant on reducing bladder contractility for OAB through inhibiting calcium/calmodulin-dependent kinase II and voltage-dependent calcium channel was also reported in a PBOO model [131,132,133]. On the contrary, the study suggested that melatonin inhibits smooth muscle contractility and may be a useful agent for overactive bladder [56].

### 9.4. Omega-3 Fatty Acid

Omega-3 fatty acid is an essential fatty acid and is recognized as the important structural component of the cell membrane. The important sources of omega-3 fatty acid are cereal production, fish, seeds, nuts and marine invertebrates [134]. Omega-3 fatty acid has anti-inflammatory and antioxidative properties [135]. A previous study supported that omega-3 fatty acid has beneficial effects in various cardiovascular diseases, neurodegenerative disease, rheumatoid arthritis and cancers [136].

In a rat PBOO model, orally administered omega-3 fatty acid significantly reduced bladder weight and fibrosis than PBOO rats. Furthermore, omega-3 fatty acid treatment meaningfully modulated the levels of the bladder CAT, SOD and MDA, as well as serum SOD and GSH-Px [58]. In another PBOO rat model, bladder tissue inflammation and fibrosis were significantly decreased after feeding with omega-3 fatty acid for 4 weeks [58]. Additionally, the expression of oxidative stress biomarkers and antioxidative defense in bladder tissue and serum were also changed. However, the results were not all consistent with what was expected [58]. Therefore, the impact of omega-3 fatty acid on lower urinary tract symptoms required more investigation to clarify [58,135].

### 9.5. Eviprostat

Eviprostat, a phytotherapeutic agent, is composed of several plant extracts, such as *Chimaphila umbellata*, *Populus tremula*, *Pulsatilla pratensis*, *Equisetum arvense*, *Tritium aestivum* and wheat germ oil. Eviprostat has been applied for LUTS in Japan and Germany [137]. Eviprostat treatment decreased urinary levels of 8-OHdG, improved oxidative stress in LUTS and BPH patients and decreased the plasma levels of MDA and 8-OHdG. Matsui et al. [64] found that Eviprostat decreased MDA and 8-OHdG levels and expressed a normalized micturition interval in a rat model of atherosclerosis-induced chronic bladder ischemia. Urinary oxidative stress markers and bladder proinflammatory cytokine levels were also significantly increased in I/R injury [47,64,138]. A retrospective study proved the efficacy of Eviprostat in the improvement of average urinary flow rate, peak urinary flow rate, prostatic volume and symptom score.

A histological study also revealed Eviprostat treatment decreased inflammation of prostatitis [139]. Clinical studies showed that Eviprostat decreases inflammation and improves the International Prostate Symptom Score (IPSS), QoL score and urinary flow rates, decreases prostatic volume and reduces inflammation in BPH patients [139]. The effect of Eviprostat attenuated DO by its anti-inflammatory effect, the downregulation of bladder muscarinic receptors and decreased reactive oxygen species [137,140,141]. It can also prevent bladder dysfunction and pathological changes, including submucosal hemorrhage, accumulation of leukocytes and edema via suppression of oxidative stress in a PBOO rat model [47,138]. The Eviprostat-mediated decrease in bladder oxidative stress and inflammation caused by PBOO may contribute to the protection of bladder function [47]. Therefore, antioxidant and anti-inflammatory activities of Eviprostat are responsible for beneficial effects observed in the treatment of PBOO.

### 9.6. Sulforaphane

Sulforaphane, a sulfur-rich compound found in cruciferous vegetables, has antioxidation; antiapoptosis, inhibiting mitochondrial dysfunction; and anticancer activities. Sulforaphane treatment prolonged micturition interval, suppressed collagen deposition and improved bladder compliance in a PBOO rat model [55,142]. Liu et al. found sulforaphane ameliorates the increase of MDA and the reduction of SOD, GSH and CAT in PBOO rats [55]. Furthermore, its antioxidative ability possibly mediated by the expression of the Nrf2 pathway regulated the cellular antioxidative responses in consistent with other studies [143,144]. Therefore, the therapeutic effect of sulforaphane inhibits excessive ROS accumulation, protects the cell organelle from oxidative damage and subsequently preserves the organ function [55,144,145].

### 9.7. Hydrogen Water

Molecular hydrogen (H_2_) has been applied to medical treatments in recent years. It can be inhaled and absorbed as a gas; it can also be injected or drank as a hydrogen-rich aqueous solution [146]. H_2_ showed a protective effect against reperfusion injury in cerebral and myocardial infarction [147,148]. Other beneficial effects in various diseases on animal models and humans are such as anti-inflammation, antiapoptosis and stimulation of energy metabolism. Though the mechanism was not well understood, the reduction of oxidative stress was speculated as a primary reason [149].

Several studies showed H_2_ has an antioxidant effect on I/R injury in neural diseases, metabolic syndrome and cardiovascular diseases [115,147,150]. In a PBOO rat model study, treatment with H_2_ water significantly improved micturition volume and PVR [52]. Oxidative stress markers, such as increased 8-OHdG levels in urinary and bladder tissue and MDA levels in bladder tissue, were diminished by the administration of H_2_ water [52]. Another PBOO rat study also revealed that decreased contractile responses of bladder detrusor muscle strips to electrical field stimulation, carbachol and KCl in obstructed rats were reversed by H_2_ water supply. Therefore, H_2_ water might protect bladder dysfunctions from PBOO via suppression of increased oxidative stress.

## 10. Problems to Be Solved in the Future

According to present studies, monotherapy using an antioxidant agent alone may not be sufficient to reduce symptoms and obtain objective data in patients with OAB. Therefore, combination therapies of antioxidants and other agents may be useful for those patients. Because it is difficult to collect bladder tissue in clinical OAB patients, animal experiments on rats and rabbits are feasible to assess the pathological mechanism of oxidative stress and the efficacy of antioxidants in OAB.

At present, biomarkers of oxidative stress, including 8-OHdG, MDA and IsoPs obtained from bladder tissue, urine and blood of OAB subjects, are essential to determine the efficacy and safety of antioxidants agents. However, the detailed pathological mechanism of biomarkers involved in OAB remains unknown. In addition, various factors involved in oxidative stress are associated with the etiology and development of bladder dysfunction caused by OAB. For example, inflammatory cytokines, immune responses, nitric oxide synthases, p62 and various growth factors also play crucial roles [151,152]. Furthermore, several transcriptional factors, including Nrf2 and NF-kB, play a pivotal role in urinary dysfunction via the cellular response to oxidative stress [59,61]. The correlation between these damaging and protective mechanisms of oxidative stress in OAB development needs to be solved in the future.

## 11. Conclusions

Current reports strongly support that oxidative stress plays an important role in the pathogenesis of storage urinary dysfunction. It has been suggested that oxidative stress is involved in OAB and LUTS, including PBOO, bladder I/R, MetS and OHD. However, the clinical relevance of ROS/RNS production and antioxidant therapies in OAB is still unclear. Most attempts to validate and exploit chronic antioxidant therapies have provided disappointing results. Identification of the specific ROS/RNS involved in DO and OAB studies is important for the development of antioxidants. The relationships between urinary dysfunction and oxidative stress biomarkers in urine, blood and bladder tissue need to be explored in further work.

## Figures and Tables

**Table 1:** Changes in oxidative stress biomarkers in bladder detrusor overactivity.

Biomarkers	Species	Changes	Sample	Animal Model		References
DNA base oxidation						
8-OHdG	Rat	Increased	Urine	PBOO		[47]
	Rabbit	Increased	Urine	PBOO		[47,48,49]
	Human	Increased	Urine	OAB		[50]
	Rabbit	Increased	Urine Plasma	PBOO		[51]
	Rabbit	Increased	Urine Plasma	PBOO		[48]
	Rat	Increased	Urine Bladder tissue	BOO		[52]
Lipid peroxidation						
MDA	Rabbit	Increased	Plasma		PBOO	[51]
	Rabbit	Increased	Plasma		PBOO	[48]
	Human Women	Increased	Plasma		OAB	[50] [53]
	Rat	Increased	Serum		BOO	[54]
	Rat	Increased	Plasma Bladder tissue		PBOO	[55]
	Rat	Increased	Bladder tissue		I/R	[56]
	Rabbit	Increased	Bladder tissue		PBOO	[57]
	Rat	Increased	Bladder tissue		PBOO	[47]
	Rat	No change	Serum Bladder tissue		PBOO	[58]
	Rat	Increased	Bladder tissue		PBOO	[52]
	Rat	Increased	Bladder tissue		PBOO	[59]
	Rat	Increased	Bladder tissue		BOO	[60]
	Rat	Increased	Bladder tissue		PBOO	[61]
F2-IsoP	Mouse	No change	Bladder tissue		PBOO	[62]
	Mouse	Increased	Bladder tissue		PBOO	[63]

Note: PBOO, partial bladder outlet obstruction; BOO, bladder outlet obstruction; OAB, overactive bladder; I/R, ischemia/reperfusion; MDA, malondialdehyde; 8-OHdG, 8-hydroxy-2-deoxyguanosine; F2-IsoP, F2-isoprostane.

**Table 2:** Summary of research findings for effects of various antioxidants.

Antioxidant	Model	Species	Sample	Changes in Oxidative Biomarkers	Changes in Antioxidants	References
EGCG	PBOO	Rat	Bladder tissue	MDA↓	CAT↑ tSOD↑ GSH-Px↑	[61]
	Type 2 diabetes	Rat	leukocytes	8-OHdG↓ MDA↓	–	[110]
CoQ10	I/R	Rabbit	Bladder tissue	-	CAT↑ SOD↑	[111]
	I/R	Rat	Bladder tissue	MDA↓	–	[112]
	PBOO	Rabbit	Bladder tissue	NT↓ DNP↓	–	[113]
Melatonin	I/R	Rat	Bladder tissue	MDA↓ MPO↓	GSH↑	[56]
	PBOO	Rabbit	Bladder tissue	MDA↓	CAT↑ SOD↑ GSH↑	[57]
Omega-3 fatty acid	PBOO PBOO	Rat Rat	Bladder tissue	MDA↑ NO↑	SOD↓	[58]
			Serum	MDA↓ NO↓	SOD↓ GSH↓	[58]
Eviprostat	PBOO	Rat	Urine	8-OHdG↓	–	[47,49]
			Bladder tissue	MDA↓	–	[47]
	I/R	Rat	Urine	8-OHdG↓	–	[64]
			Bladder tissue	MDA↓	–	[64]
	I/R	Rat	Urine	8-OHdG↓	–	[114]
Hydrogen water	PBOO	Rat	Urine bladder tissue	8-OHdG↓ MDA↓	–	[115]
	PBOO	Rat	Urine	8-OHdG↓	–	[52]
			Bladder tissue	8-OHdG↓ MDA↓	–	[52]
Sulforaphane	PBOO	Rat	Bladder tissue	MDA↓	CAT↑ SOD↑ GSH↑	[55]

Note: PBOO, partial bladder outlet obstruction; BOO, bladder outlet obstruction; BPH, benign prostatic hyperplasia; I/R, ischemia/reperfusion; MDA, malondialdehyde; 8-OHdG 8-hydroxy-2-deoxyguanosine; SOD, superoxide dismutase; CAT, catalase; GSH, glutathione; GSH-Px, glutathione peroxidase; tSOD, total superoxide dismutase; MPO, Myeloperoxidase; NO, nitric oxide; DNP, dinitrophenyl; NT, nitrotyrosine.

## Data Availability

No applicable.

## Abbreviations

ARE	antioxidant response element
α1-AR	α1- adrenoceptor
β -AR	β-adrenoceptor
BDNF	brain-derived neurotrophic factor
BPH	benign prostatic hyperplasia
CAT	catalases
COX-2	cyclooxygenase-2
CRP	C-reactive protein
DO	detrusor overactivity
EGCG	epigallocatechin-3-gallate
F2-IsoPs	F2-isoprostanes
GSH	glutathione
GSH-Px	glutathione peroxidase
HFHS	high fat high sugar
HO-1	hemeoxygenase-1
IC/PBS	interstitial cystitis/painful bladder syndrome
I/R	ischemia/reperfusion
Keap1	Kelch-like ECH-associated protein 1
LUTS	lower urinary tract symptoms
MDA	malondialdehyde
NGF	nerve growth factor
Nrf2	nuclear transcription factor E2-related factor 2
NOS	nitric oxide synthase
NOX	nicotinamide adenine dinucleotide phosphate (NADPH) oxidase
OAB	overactive bladder
OVX	ovariectomy
OHD	ovarian hormone depletion (deficiency)
8-OHdG	8-hydroxy-2′-deoxyguanosine
PBOO	partial bladder outlet obstruction
PGE2	prostaglandin E2
ROS	reactive oxygen species
SOD	superoxide dismutase
UTI	urinary tract infection

## References

[1] Przydacz M., Golabek T., Dudek P., Lipinski M., Chlosta P. (2020). Prevalence and bother of lower urinary tract symptoms and overactive bladder in Poland, an Eastern European Study. Sci. Rep..

[2] Haylen B.T., de Ridder D., Freeman R.M., Swift S.E., Berghmans B., Lee J., Monga A., Petri E., Rizk D.E., Sand P.K. (2010). An international urogynecological association (IUGA)/international continence society (ICS) joint report on the terminology for female pelvic floor dysfunction. Neurourol. Urodyn..

[3] Lapitan M.C., Chye P.L., Asia-Pacific Continence Advisory B. (2001). The epidemiology of overactive bladder among females in Asia: A questionnaire survey. Int. Urogynecol. J. Pelvic Floor Dysfunct..

[4] Moorthy P., Lapitan M.C., Quek P.L., Lim P.H. (2004). Prevalence of overactive bladder in Asian men: An epidemiological survey. BJU Int..

[5] Chuang Y.-C., Liu S.-P., Lee K.-S., Liao L., Wang J., Yoo T.K., Chu R., Sumarsono B. (2019). Prevalence of overactive bladder in China, Taiwan and South Korea: Results from a cross-sectional, population-based study. LUTS Low. Urin. Tract Symptoms.

[6] Jenks J.C. (2016). Overactive bladder in women. Nurs. Stand..

[7] Al-Zahrani A.A., Gajewski J. (2016). Urodynamic findings in women with refractory overactive bladder symptoms. Int. J. Urol. Off. J. Jpn. Urol. Assoc..

[8] Brown J.S., Vittinghoff E., Wyman J.F., Stone K.L., Nevitt M.C., Ensrud K., Grady D. (2000). Urinary Incontinence: Does it Increase Risk for Falls and Fractures?. J. Am. Geriatr. Soc..

[9] Chu F.M., Dmochowski R. (2006). Pathophysiology of overactive bladder. Am. J. Med..

[10] Yoshimura N., Kaiho Y., Miyazato M., Yunoki T., Tai C., Chancellor M.B., Tyagi P. (2008). Therapeutic receptor targets for lower urinary tract dysfunction. Naunyn-Schmiedeberg’s Arch. Pharmacol..

[11] Azadzoi K.M., Shinde V.M., Tarcan T., Kozlowski R., Siroky M.B. (2003). Increased leukotriene and prostaglandin release, and overactivity in the chronically ischemic bladder. J. Urol..

[12] Apostolidis A., Brady C.M., Yiangou Y., Davis J., Fowler C.J., Anand P. (2005). Capsaicin receptor TRPV1 in urothelium of neurogenic human bladders and effect of intravesical resiniferatoxin. Urology.

[13] Sun Y., Chai T.C. (2004). Up-regulation of P2 × 3 receptor during stretch of bladder urothelial cells from patients with interstitial cystitis. J. Urol..

[14] Yokoyama T., Nozaki K., Fujita O., Nose H., Inoue M., Kumon H. (2004). Role of c afferent fibers and monitoring of intravesical resiniferatoxin therapy for patients with idiopathic detrusor overactivity. J. Urol..

[15] Birder L.A., de Groat W.C. (2007). Mechanisms of disease: Involvement of the urothelium in bladder dysfunction. Nat. Clin. Pract. Urol..

[16] Munoz A., Smith C.P., Boone T.B., Somogyi G.T. (2011). Overactive and underactive bladder dysfunction is reflected by alterations in urothelial ATP and NO release. Neurochem. Int..

[17] Masuda H., Kihara K., Saito K., Matsuoka Y., Yoshida S., Chancellor M.B., De Groat W.C., Yoshimura N. (2008). Reactive oxygen species mediate detrusor overactivity via sensitization of afferent pathway in the bladder of anaesthetized rats. BJU Int..

[18] Azadzoi K.M., Tarcan T., Kozlowski R., Krane R.J., Siroky M.B. (1999). Overactivity and structural changes in the chronically ischemic bladder. J. Urol..

[19] Nomiya M., Yamaguchi O., Andersson K.-E., Sagawa K., Aikawa K., Shishido K., Yanagida T., Kushida N., Yazaki J., Takahashi N. (2012). The effect of atherosclerosis-induced chronic bladder ischemia on bladder function in the rat. Neurourol. Urodynamics.

[20] Chapple C.R., Nazir J., Hakimi Z., Bowditch S., Fatoye F., Guelfucci F., Khemiri A., Siddiqui E., Wagg A. (2017). Persistence and Adherence with Mirabegron versus Antimuscarinic Agents in Patients with Overactive Bladder: A Retrospective Observational Study in UK Clinical Practice. Eur. Urol..

[21] Chapple C.R., Khullar V., Gabriel Z., Muston D., Bitoun C.E., Weinstein D. (2008). The Effects of Antimuscarinic Treatments in Overactive Bladder: An Update of a Systematic Review and Meta-Analysis. Eur. Urol..

[22] Carrière I., Fourrier-Reglat A., Dartigues J.-F., Rouaud O., Pasquier F., Ritchie K., Ancelin M.-L. (2009). Drugs With Anticholinergic Properties, Cognitive Decline, and Dementia in an Elderly General Population. Arch. Intern. Med..

[23] Orasanu B., Mahajan S.T. (2013). The use of botulinum toxin for the treatment of overactive bladder syndrome. Indian J. Urol..

[24] Marcelissen T., Cornu J.-N., Antunes-Lopes T., Geavlete B., Delongchamps N.B., Rashid T., Rieken M., Rahnama’I M.S. (2018). Management of Idiopathic Overactive Bladder Syndrome: What Is the Optimal Strategy After Failure of Conservative Treatment?. Eur. Urol. Focus.

[25] Jiang Y.H., Yu W.R., Kuo H.C. (2020). Therapeutic Effect of Botulinum Toxin A on Sensory Bladder Disorders-From Bench to Bedside. Toxins.

[26] Lee Y.-C., Chuang S.-M., Lin K.-L., Chen W.-C., Lu J.-H., Chueh K.-S., Shen M.-C., Liu L.-W., Long C.-Y., Juan Y.-S. (2020). Low-Intensity Extracorporeal Shock Wave Therapy Ameliorates the Overactive Bladder: A Prospective Pilot Study. BioMed Res. Int..

[27] Sies H. (2017). Hydrogen peroxide as a central redox signaling molecule in physiological oxidative stress: Oxidative eustress. Redox Biol..

[28] Lambeth J.D. (2004). NOX enzymes and the biology of reactive oxygen. Nat. Rev. Immunol..

[29] Brand M.D. (2010). The sites and topology of mitochondrial superoxide production. Exp. Gerontol..

[30] Nocchi L., Daly D.M., Chapple C., Grundy D. (2014). Induction of oxidative stress causes functional alterations in mouse urothelium via a TRPM8-mediated mechanism: Implications for aging. Aging Cell.

[31] Nguyen T., Nioi P., Pickett C.B. (2009). The Nrf2-antioxidant response element signaling pathway and its activation by oxidative stress. J. Biol. Chem..

[32] Levonen A.-L., Landar A., Ramachandran A., Ceaser E.K., Dickinson D.A., Zanoni G., Morrow J.D., Darley-Usmar V.M. (2004). Cellular mechanisms of redox cell signalling: Role of cysteine modification in controlling antioxidant defences in response to electrophilic lipid oxidation products. Biochem. J..

[33] Liu K., Luo M., Wei S. (2019). The Bioprotective Effects of Polyphenols on Metabolic Syndrome against Oxidative Stress: Evidences and Perspectives. Oxid. Med. Cell Longev..

[34] Baird L., Yamamoto M. (2020). The Molecular Mechanisms Regulating the KEAP1-NRF2 Pathway. Mol. Cell. Biol..

[35] Hyung J.H., Ahn C.B., Il Kim B., Kim K., Je J.Y. (2016). Involvement of Nrf2-mediated heme oxygenase-1 expression in anti-inflammatory action of chitosan oligosaccharides through MAPK activation in murine macrophages. Eur. J. Pharm..

[36] Chen L., Teng H., Zhang K.Y., Skalicka-Woźniak K., Georgiev M.I., Xiao J. (2017). Agrimonolide and Desmethylagrimonolide Induced HO-1 Expression in HepG2 Cells through Nrf2-Transduction and p38 Inactivation. Front. Pharmacol..

[37] Da Costa L.A., Badawi A., El-Sohemy A. (2012). Nutrigenetics and modulation of oxidative stress. Ann. Nutr. Metab..

[38] Fridovich I. (1997). Superoxide anion radical (O2-.), superoxide dismutases, and related matters. J. Biol. Chem..

[39] Guven A., Kalorin C., Onal B., Whitbeck C., Chichester P., Kogan B.A., Levin R.M., Mannikarottu A. (2007). Novel biomarkers of bladder decompensation after partial bladder obstruction. Neurourol. Urodynamics.

[40] Zelko I.N., Mariani T.J., Folz R.J. (2002). Superoxide dismutase multigene family: A comparison of the CuZn-SOD (SOD1), Mn-SOD (SOD2), and EC-SOD (SOD3) gene structures, evolution, and expression. Free Radic. Biol. Med..

[41] Bhide A.A., Cartwright R., Khullar V., Digesu G.A. (2013). Biomarkers in overactive bladder. Int. Urogynecol. J..

[42] Hsieh J.-T., Kuo K.-L., Liu S.-H., Shi C.-S., Chang H.-C., Lin W.-C., Chou C.-T., Hsu C.-H., Liao S.-M., Wang Z.-H. (2016). Epigallocatechin Gallate Attenuates Partial Bladder Outlet Obstruction-induced Bladder Injury via Suppression of Endoplasmic Reticulum Stress-related Apoptosis—In Vivo Study. Urol..

[43] Lu B., Poirier C., Gaspar T., Gratzke C., Harrison W., Busija D., Matzuk M.M., Andersson K.-E., Overbeek P., Bishop C.E. (2008). A Mutation in the Inner Mitochondrial Membrane Peptidase 2-Like Gene (Immp2l) Affects Mitochondrial Function and Impairs Fertility in Mice1. Biol. Reprod..

[44] Soler R., Fullhase C., Lu B., Bishop C.E., Andersson K.E. (2010). Bladder dysfunction in a new mutant mouse model with increased superoxide--lack of nitric oxide?. J. Urol..

[45] Andersson K.E. (2018). Oxidative stress and its possible relation to lower urinary tract functional pathology. BJU Int..

[46] Dalle-Donne I., Rossi R., Colombo R., Giustarini D., Milzani A. (2006). Biomarkers of oxidative damage in human disease. Clin. Chem..

[47] Oka M., Fukui T., Ueda M., Tagaya M., Oyama T., Tanaka M. (2009). Suppression of Bladder Oxidative Stress and Inflammation by a Phytotherapeutic Agent in a Rat Model of Partial Bladder Outlet Obstruction. J. Urol..

[48] Lin W.-Y., Wu S.-B., Lin Y.-P., Chang P.-J., Levin R.M., Wei Y.-H. (2012). Reversing bladder outlet obstruction attenuates systemic and tissue oxidative stress. BJU Int..

[49] Matsumoto S., Hanai T., Matsui T., Oka M., Tanaka M., Uemura H. (2009). Eviprostat suppresses urinary oxidative stress in a rabbit model of partial bladder outlet obstruction and in patients with benign prostatic hyperplasia. Phytotherapy Res..

[50] Dokumacioglu E., Demiray O., Dokumacioglu A., Sahin A., Sen T.M., Cankaya S. (2018). Measuring urinary 8-hydroxy-2′-deoxyguanosine and malondialdehyde levels in women with overactive bladder. Investig. Clin. Urol..

[51] Lin W.-Y., Chen C.-S., Wu S.-B., Lin Y.-P., Levin R.M., Wei Y.-H. (2010). Oxidative stress biomarkers in urine and plasma of rabbits with partial bladder outlet obstruction. BJU Int..

[52] Miyazaki N., Yamaguchi O., Nomiya M., Aikawa K., Kimura J. (2016). Preventive Effect of Hydrogen Water on the Development of Detrusor Overactivity in a Rat Model of Bladder Outlet Obstruction. J. Urol..

[53] Rada M.P., Ciortea R., Măluţan A.M., Doumouchtsis S.K., Bucuri C.E., Clim A., Roman A., Mihu D. (2020). The profile of urinary biomarkers in overactive bladder. Neurourol. Urodynamics.

[54] Yildirim A., Başeskioğlu B., Temel H.E., Erkasap N., Yenilmez A., Uslu S., Özer C., Ozkurt M., Dönmez T. (2012). Effect of αlipoic acid and silymarin on bladder outlet obstruction. Exp. Ther. Med..

[55] Liu C., Xu H., Fu S., Chen Y., Chen Q., Cai Z., Zhou J., Wang Z. (2016). Sulforaphane Ameliorates Bladder Dysfunction through Activation of the Nrf2-ARE Pathway in a Rat Model of Partial Bladder Outlet Obstruction. Oxidative Med. Cell. Longev..

[56] Sener G., Sehirli A.O., Paskaloglu K., Dulger G.A., Alican I. (2003). Melatonin treatment protects against ischemia/reperfusion-induced functional and biochemical changes in rat urinary bladder. J. Pineal Res..

[57] Onur R., Tasdemir C., Seckin D., Ilhan N., Kutlu S., Akpolat N. (2008). Combined Use of Melatonin and Terazosin Restores Bladder Contractility in Rabbits With Partial Outlet Obstruction. Urology.

[58] Firat F., Uluocak N., Erdemir F., Atilgan D., Markoc F., Parlaktas B.S., Yasar A. (2016). Evaluation of the effects of omega-3 & interferon alpha-2b administration on partial bladder outlet obstruction in a rat model. Indian J. Med Res..

[59] Sezginer E.K., Yilmaz-Oral D., Lokman U., Nebioglu S., Aktan F., Gur S. (2019). Effects of varying degrees of partial bladder outlet obstruction on urinary bladder function of rats: A novel link to inflammation, oxidative stress and hypoxia. LUTS: Low. Urin. Tract Symptoms.

[60] Sun J., Shen W., An W., Li Q., Qiu S., Jiang S. (2017). A Chinese Medicine Formula “Xian-Jia-Tang” for Treating Bladder Outlet Obstruction by Improving Urodynamics and Inhibiting Oxidative Stress through Potassium Channels. Evidence-Based Complement. Altern. Med..

[61] Gu M., Liu C., Wan X., Yang T., Chen Y., Zhou J., Chen Q., Wang Z. (2018). Epigallocatechin Gallate Attenuates Bladder Dysfunction via Suppression of Oxidative Stress in a Rat Model of Partial Bladder Outlet Obstruction. Oxidative Med. Cell. Longev..

[62] Stephany H.A., Strand D., Ching C.B., Tanaka S.T., Milne G.L., Cajaiba M.M., Thomas J.C., Pope J.C., Adams M.C., Brock J.W. (2013). Chronic Cyclic Bladder Over Distention Up-Regulates Hypoxia Dependent Pathways. J. Urol..

[63] Clayton D.B., Stephany H.A., Ching C.B., Rahman S.A., Tanaka S.T., Thomas J.C., Pope J.C., Adams M.C., Brock J.W., Clark P.E. (2014). F 2 -Isoprostanes as a Biomarker of Oxidative Stress in the Mouse Bladder. J. Urol..

[64] Matsui T., Oka M., Fukui T., Tanaka M., Oyama T., Sagawa K., Nomiya M., Yamaguchi O. (2012). Suppression of bladder overactivity and oxidative stress by the phytotherapeutic agent, Eviprostat, in a rat model of atherosclerosis-induced chronic bladder ischemia. Int. J. Urol..

[65] Milne G.L., Yin H., Hardy K.D., Davies S.S., Roberts L.J. (2011). Isoprostane generation and function. Chem. Rev..

[66] Dambros M., Dambros M.C., Lorenzetti F., Dassen E.L., van Koeveringe G.A. (2014). The use of hypochlorous acid as a model for investigating bladder overactivity. Int. Braz. J. Urol..

[67] Tseng C.H. (2013). Benign prostatic hyperplasia is a significant risk factor for bladder cancer in diabetic patients: A population-based cohort study using the National Health Insurance in Taiwan. BMC Cancer.

[68] Callaghan C.M., Johnson A., Neumann P., Leggett R.E., Schuler C., Levin R.M. (2013). The effect of partial outlet obstruction on calpain and phospholipase-2 activities: Analyzed by severity and duration. Mol. Cell. Biochem..

[69] Zhao Y., Levin S.S., Wein A.J., Levin R.M. (1997). Correlation of ischemia/reperfusion or partial outlet obstruction-induced spectrin proteolysis by calpain with contractile dysfunction in rabbit bladder. Urology.

[70] Kim J.C., Yoo J.S., Park E.Y., Hong S.H., Seo S.I., Hwang T.-K. (2008). Muscarinic and purinergic receptor expression in the urothelium of rats with detrusor overactivity induced by bladder outlet obstruction. BJU Int..

[71] Azadzoi K.M., Pontari M., Vlachiotis J., Siroky M.B. (1996). Canine bladder blood flow and oxygenation: Changes induced by filling, contraction and outlet obstruction. J. Urol..

[72] Koritsiadis G., Stravodimos K., Koutalellis G., Agrogiannis G., Koritsiadis S., Lazaris A., Constantinides C. (2008). Immunohistochemical estimation of hypoxia in human obstructed bladder and correlation with clinical variables. BJU Int..

[73] Callaghan C.M., Schuler C., Leggett R.E., Levin R.M. (2013). Effect of severity and duration of bladder outlet obstruction on catalase and superoxide dismutase activity. Int. J. Urol..

[74] Sun S., Bai Y., Yang H., Yang H.W. (2015). Investigation on lower urinary tract symptoms (LUTS) in elderly patients with prostate cancer (PC) received endocrine therapy. Arch. Gerontol. Geriatr..

[75] Pinggera G.-M., Mitterberger M., Steiner E., Pallwein L., Frauscher F., Aigner F., Bartsch G., Strasser H. (2008). Association of lower urinary tract symptoms and chronic ischaemia of the lower urinary tract in elderly women and men: Assessment using colour Doppler ultrasonography. BJU Int..

[76] Alexandre E.C., Calmasini F.B., De Oliveira M.G., Silva F.H., Da Silva C.P., André D.M., Leonardo F.C., Delbin M.A., Antunes E. (2016). Chronic treatment with resveratrol improves overactive bladder in obese mice via antioxidant activity. Eur. J. Pharmacol..

[77] Juan Y.-S., Li S., Levin R.M., Kogan B.A., Schuler C., Leggett R.E., Huang C.-H., Mannikarottu A. (2009). The Effect of Ischemia/Reperfusion on Rabbit Bladder—Role of Rho-kinase and Smooth Muscle Regulatory Proteins. Urology.

[78] Andreadou I., Iliodromitis E.K., Lazou A., Görbe A., Giricz Z., Schulz R., Ferdinandy P. (2017). Effect of hypercholesterolaemia on myocardial function, ischaemia-reperfusion injury and cardioprotection by preconditioning, postconditioning and remote conditioning. Br. J. Pharmacol..

[79] Andersson K.E., Boedtkjer D.B., Forman A. (2017). The link between vascular dysfunction, bladder ischemia, and aging bladder dysfunction. Ther. Adv. Urol..

[80] De Nunzio C., Cindolo L., Gacci M., Pellegrini F., Carini M., Lombardo R., Franco G., Tubaro A. (2014). Metabolic Syndrome and Lower Urinary Tract Symptoms in Patients With Benign Prostatic Enlargement: A Possible Link to Storage Symptoms. Urology.

[81] Nomiya M., Sagawa K., Yazaki J., Takahashi N., Kushida N., Haga N., Aikawa K., Matsui T., Oka M., Fukui T. (2011). Increased bladder activity is associated with elevated oxidative stress markers and proinflammatory cytokines in a rat model of atherosclerosis-induced chronic bladder ischemia. Neurourol. Urodynamics.

[82] Nomiya M., Andersson K.E., Yamaguchi O. (2015). Chronic bladder ischemia and oxidative stress: New pharmacotherapeutic targets for lower urinary tract symptoms. Int. J. Urol..

[83] Punthakee Z., Goldenberg R., Katz P., Diabetes Canada Clinical Practice Guidelines Expert Committee (2018). Definition, Classification and Diagnosis of Diabetes, Prediabetes and Metabolic Syndrome. Can. J. Diabetes.

[84] Hong G.S., Shim B.S., Chung W.S., Yoon H. (2010). Correlation between Metabolic Syndrome and Lower Urinary Tract Symptoms of Males and Females in the Aspect of Gender-Specific Medicine: A Single Institutional Study. Korean J. Urol..

[85] Uzun H., Zorba O.U. (2012). Metabolic syndrome in female patients with overactive bladder. Urology.

[86] Bunn F., Kirby M., Pinkney E., Cardozo L., Chapple C., Chester K., Cruz F., Haab F., Kelleher C., Milsom I. (2015). Is there a link between overactive bladder and the metabolic syndrome in women? A systematic review of observational studies. Int. J. Clin. Pr..

[87] He Q., Wang Z., Liu G., Daneshgari F., MacLennan G.T., Gupta S. (2016). Metabolic syndrome, inflammation and lower urinary tract symptoms: Possible translational links. Prostate Cancer Prostatic Dis..

[88] Peyronnet B., Mironska E., Chapple C., Cardozo L., Oelke M., Dmochowski R., Amarenco G., Gamé X., Kirby R., Van Der Aa F. (2019). A Comprehensive Review of Overactive Bladder Pathophysiology: On the Way to Tailored Treatment. Eur. Urol..

[89] Jankovic A., Korac A., Buzadzic B., Stancic A., Otasevic V., Ferdinandy P., Daiber A., Korac B. (2016). Targeting the NO/superoxide ratio in adipose tissue: Relevance to obesity and diabetes management. Br. J. Pharmacol..

[90] Leiria L.O., Silva F.H., Davel A., Alexandre E.C., Calixto M.C., De Nucci G., Mónica F.Z., Antunes E. (2014). The Soluble Guanylyl Cyclase Activator BAY 60-2770 Ameliorates Overactive Bladder in Obese Mice. J. Urol..

[91] Rolo A.P., Palmeira C.M. (2006). Diabetes and mitochondrial function: Role of hyperglycemia and oxidative stress. Toxicol. Appl. Pharm..

[92] Wallace D.C. (2005). A mitochondrial paradigm of metabolic and degenerative diseases, aging, and cancer: A dawn for evolutionary medicine. Annu. Rev. Genet..

[93] Chung S.D., Chien C.T., Yu H.J. (2013). Alterations in peripheral purinergic and muscarinic signaling of rat bladder after long-term fructose-induced metabolic syndrome. Eur. J. Nutr..

[94] Lee Y.-L., Lin K.-L., Wu B.-N., Chuang S.-M., Wu W.J., Lee Y.-C., Ho W.-T., Juan Y.-S. (2018). Epigallocatechin-3-gallate alleviates bladder overactivity in a rat model with metabolic syndrome and ovarian hormone deficiency through mitochondria apoptosis pathways. Sci. Rep..

[95] Furukawa S., Fujita T., Shimabukuro M., Iwaki M., Yamada Y., Nakajima Y., Nakayama O., Makishima M., Matsuda M., Shimomura I. (2004). Increased oxidative stress in obesity and its impact on metabolic syndrome. J. Clin. Investig..

[96] Robinson D., Cardozo L.D. (2003). The role of estrogens in female lower urinary tract dysfunction. Urology.

[97] Cardozo L., Lose G., McClish D., Versi E. (2004). A systematic review of the effects of estrogens for symptoms suggestive of overactive bladder. Acta Obs. Gynecol. Scand..

[98] Nappi R.E., Palacios S., Panay N., Particco M., Krychman M.L. (2016). Vulvar and vaginal atrophy in four European countries: Evidence from the European REVIVE Survey. Climacteric J. Int. Menopause Soc..

[99] Batra S.C., Iosif C.S. (1987). Progesterone receptors in the female lower urinary tract. J. Urol..

[100] Blakeman P.J., Hilton P., Bulmer J.N. (2000). Oestrogen and progesterone receptor expression in the female lower urinary tract, with reference to oestrogen status. BJU Int..

[101] Cheng C.L., Li J.R., Lin C.H., de Groat W.C. (2016). Positive association of female overactive bladder symptoms and estrogen deprivation: A nationwide population-based cohort study in Taiwan. Medicine.

[102] Krause M., Wheeler T.L., Snyder T.E., Richter H.E. (2009). Local Effects of Vaginally Administered Estrogen Therapy: A Review. J. Pelvic Med. Surg..

[103] Juan Y.-S., Chuang S.-M., Long C.-Y., Chen C.-H., Levin R.M., Liu K.-M. (2012). Neuroprotection of green tea catechins on surgical menopause-induced overactive bladder in a rat model. Menopause.

[104] Juan Y.-S., Huang C.-H., Lee Y.-L., Long C.-Y., Wu T.-H., Chang W.-C., Levin R.M., Liu K.-M. (2012). Green tea catechins decrease oxidative stress in surgical menopause-induced overactive bladder in a rat model. BJU Int..

[105] Weiderpass E., A Baron J., Adami H.-O., Magnusson C., Lindgren A., Bergström R., Correia N., Persson I. (1999). Low-potency oestrogen and risk of endometrial cancer: A case-control study. Lancet.

[106] Iosif C.S., Bekassy Z. (1984). Prevalence of genito-urinary symptoms in the late menopause. Acta Obs. Gynecol. Scand..

[107] Lin W.-Y., Li S., Leggett R., Strassner J., Sokol R., Schuler C., Juan Y.-S., Javed Z., Kogan B., Levin R.M. (2009). Estrogen administration attenuates bladder outlet obstruction induced oxidative stress in the female rabbit. Neurourol. Urodynamics.

[108] Chuang S.-M., Long C.-Y., Lin R.-J., Liu K.-M., Levin R.M., Chang C.-Y., Ho Y.-W., Wu W.J., Chang W.-C., Juan Y.-S. (2013). Protective effects of estrogen on ischemia/reperfusion–induced bladder dysfunction in female rabbits. Menopause.

[109] Roy J., Galano J.M., Durand T., Le Guennec J.Y., Lee J.C. (2017). Physiological role of reactive oxygen species as promoters of natural defenses. FASEB J..

[110] Uchiyama Y., Suzuki T., Mochizuki K., Goda T. (2013). Dietary supplementation with a low dose of (-)-epigallocatechin-3-gallate reduces pro-inflammatory responses in peripheral leukocytes of non-obese type 2 diabetic GK rats. J. Nutr. Sci. Vitam..

[111] Juan Y.-S., Hydery T., Mannikarottu A., Kogan B., Schuler C., Leggett R.E., Lin W.-Y., Huang C.-H., Levin R.M. (2008). Coenzyme Q10 protect against ischemia/reperfusion induced biochemical and functional changes in rabbit urinary bladder. Mol. Cell. Biochem..

[112] Kim J.W., Jang H.A., Bae J.H., Lee J.G. (2013). Effects of coenzyme Q10 on bladder dysfunction induced by chronic bladder ischemia in a rat model. J. Urol..

[113] Juan Y.-S., Levin R.M., Chuang S.M., Hydery T., Li S., Kogan B., Schuler C., Mannikarottu A., Huang C.-H. (2008). The Beneficial Effect of Coenzyme Q10 and Lipoic Acid on Obstructive Bladder Dysfunction in the Rabbit. J. Urol..

[114] Matsumoto S., Hanai T., Shimizu N., Sugimoto K., Uemura H. (2010). Effect of edaravone on ischemia/reperfusion injury in rat urinary bladder—Changes in smooth muscle cell phenotype and contractile function. Aktuelle Urol..

[115] Kajiyama S., Hasegawa G., Asano M., Hosoda H., Fukui M., Nakamura N., Kitawaki J., Imai S., Nakano K., Ohta M. (2008). Supplementation of hydrogen-rich water improves lipid and glucose metabolism in patients with type 2 diabetes or impaired glucose tolerance. Nutr. Res..

[116] Chu C., Deng J., Man Y., Qu Y. (2017). Green Tea Extracts Epigallocatechin-3-gallate for Different Treatments. Biomed. Res. Int..

[117] Miyata Y., ScienMatsuoces T., Araki K., Nakamura Y., Sagara Y., Ohba K., Sakai H. (2018). Anticancer Effects of Green Tea and the Underlying Molecular Mechanisms in Bladder Cancer. Medicines.

[118] Miyata Y., Shida Y., Hakariya T., Sakai H. (2019). Anti-Cancer Effects of Green Tea Polyphenols Against Prostate Cancer. Molecules.

[119] Xing L., Zhang H., Qi R., Tsao R., Mine Y. (2019). Recent Advances in the Understanding of the Health Benefits and Molecular Mechanisms Associated with Green Tea Polyphenols. J. Agric. Food Chem..

[120] Liu Z., Chen R., Jiang Y., Yang Y., He L., Luo C., Dong J., Rong L. (2019). A meta-analysis of serum osteocalcin level in postmenopausal osteoporotic women compared to controls. BMC Musculoskelet. Disord..

[121] Chung J.E., Kurisawa M., Kim Y.J., Uyama H., Kobayashi S. (2004). Amplification of antioxidant activity of catechin by polycondensation with acetaldehyde. Biomacromolecules.

[122] Tipoe G.L., Leung T.M., Hung M.W., Fung M.L. (2007). Green tea polyphenols as an anti-oxidant and anti-inflammatory agent for cardiovascular protection. Cardiovasc. Hematol. Disord. Drug Targets.

[123] Yuan T., Yang T., Chen H., Fu D., Hu Y., Wang J., Yuan Q., Yu H., Xu W., Xie X. (2019). New insights into oxidative stress and inflammation during diabetes mellitus-accelerated atherosclerosis. Redox Biol..

[124] Levites Y., Weinreb O., Maor G., Youdim M.B., Mandel S. (2001). Green tea polyphenol (-)-epigallocatechin-3-gallate prevents N-methyl-4-phenyl-1,2,3,6-tetrahydropyridine-induced dopaminergic neurodegeneration. J. Neurochem..

[125] Mandel S.A., Avramovich-Tirosh Y., Reznichenko L., Zheng H., Weinreb O., Amit T., Youdim M.B. (2005). Multifunctional Activities of Green Tea Catechins in Neuroprotection. Neurosignals.

[126] Qanungo S., Das M., Haldar S., Basu A. (2005). Epigallocatechin-3-gallate induces mitochondrial membrane depolarization and caspase-dependent apoptosis in pancreatic cancer cells. Carcinogenesis.

[127] Coyle C.H., Philips B.J., Morrisroe S.N., Chancellor M.B., Yoshimura N. (2008). Antioxidant effects of green tea and its polyphenols on bladder cells. Life Sci..

[128] Moon Y., Lee K.H., Park J.H., Geum D., Kim K. (2005). Mitochondrial membrane depolarization and the selective death of dopaminergic neurons by rotenone: Protective effect of coenzyme Q10. J. Neurochem..

[129] Mancuso M., Orsucci D., Volpi L., Calsolaro V., Siciliano G. (2010). Coenzyme Q10 in neuromuscular and neurodegenerative disorders. Curr. Drug Targets.

[130] Bubenik G.A., Blask D.E., Brown G.M., Maestroni G.J., Pang S.F., Reiter R.J., Viswanathan M., Zisapel N. (1998). Prospects of the Clinical Utilization of Melatonin. Neurosignals.

[131] Tordjman S., Chokron S., Delorme R., Charrier A., Bellissant E., Jaafari N., Fougerou C. (2017). Melatonin: Pharmacology, Functions and Therapeutic Benefits. Curr. Neuropharmacol..

[132] Han J.H., Chang I.H., Myung S.C., Lee M.Y., Kim W.Y., Lee S.Y., Lee S.Y., Lee S.W., Kim K.D. (2012). A Novel Pathway Underlying the Inhibitory Effects of Melatonin on Isolated Rat Urinary Bladder Contraction. Korean J. Physiol. Pharmacol..

[133] Semercioz A., Onur R., Ayar A., Orhan I. (2004). The inhibitory role of melatonin on isolated guinea-pig urinary bladder: An endogenous hormone effect. BJU Int..

[134] Shahidi F., Ambigaipalan P. (2018). Omega-3 Polyunsaturated Fatty Acids and Their Health Benefits. Annu. Rev. Food Sci. Technol..

[135] Farooqui A.A. (2012). n-3 fatty acid-derived lipid mediators in the brain: New weapons against oxidative stress and inflammation. Curr. Med. Chem..

[136] Jia X., Kohli P., Virani S.S. (2019). Omega-3 Fatty Acid and Cardiovascular Outcomes: Insights from Recent Clinical Trials. Curr. Atheroscler. Rep..

[137] Kobayashi M., Nomura M., Nishii H., Matsumoto S., Fujimoto N., Matsumoto T. (2008). Effect of eviprostat on bladder overactivity in an experimental cystitis rat model. Int. J. Urol..

[138] Kawai Y., Oka M., Kyotani J., Oyama T., Matsumoto S., Kakizaki H. (2013). Effect of the phytotherapeutic agent eviprostat on the bladder in a rat model of bladder overdistension/emptying. Neurourol. Urodynamics.

[139] Ishigooka M., Hashimoto T., Hayami S., Tomaru M., Nakada T., Mitobe K. (1995). Clinical and retrospective evaluation of eviprostat: A non-hormonal and non-neuropharmacological agent for benign prostatic hyperplasia. Int. Urol. Nephrol..

[140] Nasrin S., Masuda E., Kugaya H., Ito Y., Yamada S. (2013). Improvement by phytotherapeutic agent of detrusor overactivity, down-regulation of pharmacological receptors and urinary cytokines in rats with cyclophosphamide induced cystitis. J. Urol..

[141] Oka M., Tachibana M., Noda K., Inoue N., Tanaka M., Kuwabara K. (2007). Relevance of anti-reactive oxygen species activity to anti-inflammatory activity of components of Eviprostat®, a phytotherapeutic agent for benign prostatic hyperplasia. Phytomedicine.

[142] Liu C., Wan X., Gu M., Chen Y., Cai Z., Zhou J., Chen Q., Wang Z. (2019). Effect of Sulforaphane on Bladder Compliance in a Rat Model of Partial Bladder Outlet Obstruction. Oxidative Med. Cell. Longev..

[143] Bai Y., Cui W., Xin Y., Miao X., Barati M.T., Zhang C., Chen Q., Tan Y., Cui T., Zheng Y. (2013). Prevention by sulforaphane of diabetic cardiomyopathy is associated with up-regulation of Nrf2 expression and transcription activation. J. Mol. Cell. Cardiol..

[144] Lin C.-F., Chueh T.-H., Chung C.-H., Chung S.-D., Chang T.-C., Chien C.-T. (2020). Sulforaphane improves voiding function via the preserving mitochondrial function in diabetic rats. J. Formos. Med Assoc..

[145] Tai H.C., Chung S.D., Chien C.T., Yu H.J. (2016). Sulforaphane Improves Ischemia-Induced Detrusor Overactivity by Downregulating the Enhancement of Associated Endoplasmic Reticulum Stress, Autophagy, and Apoptosis in Rat Bladder. Sci. Rep..

[146] Iida A., Nosaka N., Yumoto T., Knaup E., Naito H., Nishiyama C., Yamakawa Y., Tsukahara K., Terado M., Sato K. (2016). The Clinical Application of Hydrogen as a Medical Treatment. Acta Med. Okayama.

[147] Ohsawa I., Ishikawa M., Takahashi K., Watanabe M., Nishimaki K., Yamagata K., Katsura K.-I., Katayama Y., Asoh S., Ohta S. (2007). Hydrogen acts as a therapeutic antioxidant by selectively reducing cytotoxic oxygen radicals. Nat. Med..

[148] Hayashida K., Sano M., Ohsawa I., Shinmura K., Tamaki K., Kimura K., Endo J., Katayama T., Kawamura A., Kohsaka S. (2008). Inhalation of hydrogen gas reduces infarct size in the rat model of myocardial ischemia–reperfusion injury. Biochem. Biophys. Res. Commun..

[149] Ohta S. (2014). Molecular hydrogen as a preventive and therapeutic medical gas: Initiation, development and potential of hydrogen medicine. Pharmacol. Ther..

[150] Fu Y., Ito M., Fujita Y., Ito M., Ichihara M., Masuda A., Suzuki Y., Maesawa S., Kajita Y., Hirayama M. (2009). Molecular hydrogen is protective against 6-hydroxydopamine-induced nigrostriatal degeneration in a rat model of Parkinson’s disease. Neurosci. Lett..

[151] Lin W.-Y., Hsieh C.C., Yang T.-Y., Chen M.-L., Huang L.Y., Lin Y.-P., Chang P.-J., Levin R.M., Wei Y.-H. (2014). Transient Increase in Circulating Myeloid-Derived Suppressor Cells after Partial Bladder Outlet Obstruction. J. Urol..

[152] Lin W.Y., Lin Y.P., Levin R.M., Chen M.L. (2017). The relevance of immune responses to partial bladder outlet obstruction and reversal. Neurourol. Urodyn..

